# Erythrocyte-Coated Nanoparticles Block Cytotoxic Effects of Group B *Streptococcus* β-Hemolysin/Cytolysin

**DOI:** 10.3389/fped.2019.00410

**Published:** 2019-11-01

**Authors:** Jenny Koo, Tamara Escajadillo, Liangfang Zhang, Victor Nizet, Shelley M. Lawrence

**Affiliations:** ^1^Division of Neonatal-Perinatal Medicine, Department of Pediatrics, University of California, San Diego, La Jolla, CA, United States; ^2^Collaborative to Halt Antibiotic-Resistant Microbes (CHARM), Division of Host-Microbe Systems and Therapeutics, Department of Pediatrics, University of California, San Diego, La Jolla, CA, United States; ^3^Department of Nanoengineering, University of California, San Diego, La Jolla, CA, United States; ^4^Moores Cancer Center, University of California, San Diego, La Jolla, CA, United States; ^5^Skaggs School of Pharmacy and Pharmaceutical Sciences, University of California, San Diego, La Jolla, CA, United States

**Keywords:** Group B *Streptococcus*, nanoparticle, nanosponge, neonatal sepsis, neonates, adults, biomimetic, beta-hemolysin/cytolysin

## Abstract

Group B *Streptococcus* (GBS) emerged as a leading cause of invasive infectious disease in neonates in the 1970s, but has recently been identified as an escalating public health threat in non-pregnant adults, particularly those of advanced aged or underlying medical conditions. GBS infection can rapidly develop into life-threatening disease despite prompt administration of effective antibiotics and initiation of state-of-the-art intensive care protocols and technologies due to deleterious bacterial virulence factors, such as the GBS pore-forming toxin β-hemolysin/cytolysin (β-H/C). β-H/C is known to have noxious effects on a wide range of host cells and tissues, including lung epithelial cell injury, blood brain barrier weakening, and immune cell apoptosis. Neonatal and adult survivors of GBS infection are at a high risk for substantial long-term health issues and neurologic disabilities due to perturbations in organ systems caused by bacterial- and host- mediated inflammatory stressors. Previously engineered anti-virulence inhibitors, such as monoclonal antibodies and small molecular inhibitors, generally require customized design for each different pathogenic toxin and do not target deleterious host pro-inflammatory responses that may cause organ injury, septic shock, or death. By simply wrapping donor red blood cells (RBCs) around polymeric cores, we have created biomimetic “nanosponges.” Because nanoparticles retain the same repertoire of cell membrane receptors as their host cell, they offer non-specific and all-purpose toxin decoy strategies with a broad ability to sequester and neutralize various bacterial toxins and host pro-inflammatory chemokines and cytokines to attenuate the course of infectious disease. This proof-of-concept study successfully demonstrated that intervention with nanosponges reduced the hemolytic activity of live GBS and stabilized β-H/C in a dose-dependent manner. Nanosponge treatment also decreased lung epithelial and macrophage cell death following exposure to live GBS bacteria and stabilized β-H/C, improved neutrophil killing of GBS, and diminished GBS-induced macrophage IL-1β production. Our results, therefore, suggest biomimetic nanosponges provide a titratable detoxification therapy that may provide a first-in-class treatment option for GBS infection by sequestering and inhibiting β-H/C activity.

## Introduction

Group B *Streptococcus* (GBS) is the leading cause of neonatal early-onset sepsis (EOS) with an incidence of 0.34–0.37 per 1,000 live births (1). Nearly one-third of women of child-bearing age are asymptomatic carriers of the bacterium, which can colonize up to half of infants during the birthing process without appropriate empiric intrapartum GBS prophylaxis (1, 2). Although mortality has greatly decreased over the last few decades, an estimated 30% of very low birthweight (VLBW, <1,500 g at birth) preterm and 2–3% of term infants will die from GBS EOS due to gestational age-dependent impairments of humoral immunity and primary reliance on developmentally immature innate immune responses (1, 3).

Conversely, non-pregnant adults account for 90% of the estimated 1,660 annual deaths attributable to GBS infection (4). Nearly all cases (95%) occur in persons with at least one comorbidity, including obesity (53.9%) and diabetes (43.2%) (5). In 2016, an estimated 27,729 GBS cases were reported in the U.S. (5), with 94.6% of cases requiring hospitalization, 27.3% necessitating admission to an intensive care unit, and 5.6% resulting in death (5, 6). Alarmingly, rates of invasive GBS infection roughly tripled in the U.S. between 1990 and 2016 (5, 6).

GBS exhibits pathogenicity against vulnerable populations, such as infants, the elderly, and adults with comorbidities, due to the expression of several virulence factors that exploit host susceptibilities. Amongst the most important GBS virulence factors, the secreted β-hemolysin/cytolysin (β-H/C) toxin stands out due to its broad range of host cell targets (7, 8). A pore-forming toxin expressed in more than 99% of GBS strains, β-H/C is responsible for the trademark ring of hemolysis around GBS colonies on blood agar plates and its linkage to a phenotype of orange pigmentation (9). The *cylE* gene is both essential and sufficient for β-H/C activity (8). Due to the toxin's non-specific affinity for the lipid bilayer of cell membranes, β-H/C contributes to penetration of tissue barriers and inflammatory injury in GBS invasive disease syndromes such as meningitis, infections of skin and soft tissues, osteomyelitis, bacteremia, endocarditis, arthritis, and urosepsis in adults (4, 6), as well as pneumonia, bacteremia, and/or meningitis in neonatal patients (10). Because β-H/C is sequestered and inhibited by the lipid-rich primary component of surfactant, dipalmotyl phophatidylcholine (DPPC), surfactant-deficient preterm and very low birth weight (VLBW) neonates have the highest risks for GBS pneumonia and bacteremia (11, 12).

Neutrophils are essential components of innate immunity, as they are the first line of defense against pathogenic organisms and comprise the largest number of innate immune cells. Neonatal neutrophils have well-documented reductions of neutrophil storage pools and functional deficiencies in chemotaxis, transmigration, and neutrophil extracellular trap (NET) formation (1, 13). Moreover, poorly regulated immune responses during early sepsis may increase the neonate's risk for mortality and long-term morbidity (3, 7). Similarly, adults with obesity and type 2 diabetes have impaired neutrophil function with a lower stimulation index, impaired chemotaxis, and enhanced free radical production compared to metabolically healthy individuals, which may increase their chance for infection and heighten morbidity and mortality risks (14, 15).

Current strategies for reducing the incidence of neonatal GBS early-onset sepsis involve the administration of intrapartum antibiotic prophylaxis (IAP) to GBS-colonized pregnant, laboring mothers (16). Although neonatal mortality from GBS EOS declined by more than 80% following enactment of the 1996 CDC perinatal GBS prevention guidelines, the use of maternal and neonatal empiric antibiotics has risen to levels never before encountered. Moreover, further reductions in the incidence of GBS EOS have not been observed in the last two decades (1, 3). In neonates and adults, GBS isolates with increasing minimum inhibitory concentrations (MICs) to penicillin and ampicillin have been reported in the United States and Japan (1). The proportion of GBS isolates resistant to erythromycin and clindamycin is also steadily increasing. This trend is alarming for adults and penicillin allergic patients with skin and soft tissue infections as clindamycin is considered the first-line antimicrobial agent (5, 17). Adjuvant therapies that target pathogen toxicity and host responses must, therefore, be considered.

**Figure 1:**
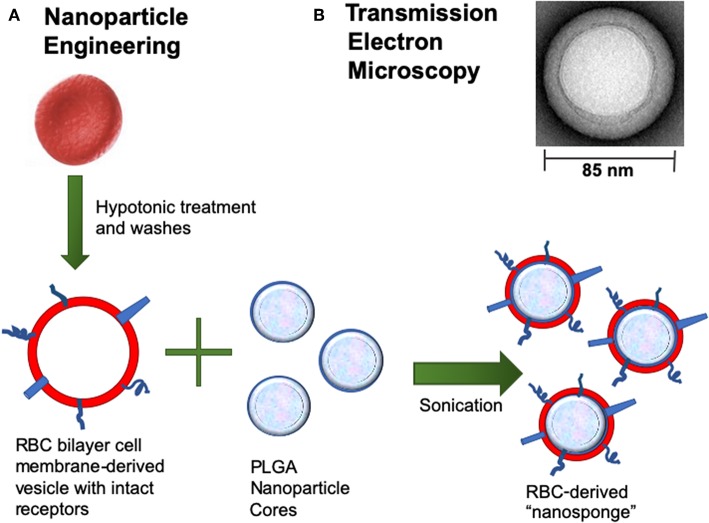
**(A)** Nanoparticle Engineering: schematic demonstrating fusion of a RBC-derived ghost membrane vesicle and PGLA core to create nanosponge therapeutics. **(B)** Representative zoomed-in image of a single toxin-absorbed nanosponge examined with transmission electron microscopy (TEM). The sample was negatively stained with uranyl acetate prior to TEM imaging.

Recent advances in nanotechnology and biomimetics has enabled the engineering of cell membrane-coated nanoparticles, which can function as biologic decoys to sequester and inhibit pathogen toxins (Figure 1). Our group has studied the function of biomimetic nanoparticles, generated by wrapping natural cell membranes derived from human erythrocytes around poly-lactico-glycolic acid (PLGA) cores, and are termed “nanosponges” or human red blood cell nanosponges (hRBC-NS) (18). Because the nanoparticles retain the same repertoire of cell membrane receptors as their host cell, they offer a multifaceted toxin decoy strategy with broad ability to sequester and neutralize various pore-forming toxins (PFT), endotoxins, and proinflammatory cytokines, regardless of their molecular structure and source. The inner polymeric core is essential for RBC membrane stabilization, enabling their prolonged half-life in the bloodstream to facilitate maximum toxin absorption (18). Nanoparticles are biodegradable, bio-compatible, and widely applicable (19–21). *In vitro* and *in vivo* studies have demonstrated complete inhibition of PFT-induced hemolysis by human-derived nanosponges for α-hemolysin of methicillin-resistant *Staphylococcus aureus* (22), listeriolysin O of *Listeria monocytogenes* (22), and streptolysin O of group A *Streptococcus* (21).

In this study, we explored the potential for hRBC-NS to specifically inhibit GBS β-H/C toxin associated cellular toxicities. Employing live GBS bacterial strains and cell-free stabilized β-H/C preparations, we found hRBC-NS effectively inhibited β-H/C mediated hemolysis, immune cell death, and lung epithelial cell culture death. This proof-of-principle study suggests the merit of future translational studies to assess the therapeutic role of hRBC-NS in neonatal and adult GBS disease.

## Methods

### Generation of Human Red Blood Cell-Coated Nanosponges (hRBC-NS)

hRBC-NS were created as previously described (18). Briefly, PLGA-COOH 0.66 dl/g was dissolved in acetone at a concentration of 5 mg/ml, then dispersed in equal volume water. Acetone was allowed to evaporate. Human RBCs from health donors are washed in PBS three times via centrifugation at 700 × g for 8 min. RBC membrane ghosts were prepared using hypotonic treatment, washed, and centrifuged at 16,000 rpm for 10 min at 4°C. RBC membrane protein was quantified using BCA assay for total protein content. Membranes were adhered to PLGA cores via sonication for 3 min at a protein-to-core ratio of 1:4. The nanosponges were spun down and resuspended in 5% sucrose diH2O at a final concentration 5–10 mg/ml for use, and stored at 4°C for no longer than 5 days or −80°C for no more than 30 days. Unless otherwise stated, the treatment concentration of hRBC-NS is 500 μg/mL for all *in vitro* experiments.

### GBS Bacterial Strains and Prep

GBS bacterial strains used in this experiment included NCTC (serotype 3), COH1 (serotype 3), A909 (serotype 1a). All bacteria are grown to mid-log phase at OD600 = 0.4 (~2 × 10^8^/ml CFU) in Todd Hewitt Broth (THB). For assays, the bacteria are spun down and resuspended in serum-free Roswell Park Memorial Institute medium (RPMI).

### β-H/C Containing GBS Supernatant

Overnight liquid cultures of GBS were subcultured at 1:20 into final volume of 500 mL and grown to mid-log phase of OD600 = 0.4. The bacteria were pelleted and washed, then resuspended in 20 ml PBS with 3% Tween 80, 1% starch, and 1% glucose for stabilization of β-H/C toxin. The solution was incubated for 1 h at 37°C, then the bacteria is pelleted, and the supernatant filtered through 0.22 μm syringe filter. The supernatant was mixed at a 1:1 volume of ice-cold methanol to precipitate out the toxin, incubated at −20°C for 1 h. Toxins were spun down and resuspended in 1 ml PBS. All centrifuge spins were done at 3,000 × g for 10 min.

### Hemolysis Assay

Human venous whole blood was collected in heparinized tubes. Red blood cells (RBC) are washed in PBS three times via centrifugation at 700 × g for 8 min. The RBC pellet was then resuspended to achieve 5% concentration by volume. GBS at 8 × 10^6^ CFU or 1 μl β-H/C+ extract is used to infect 100 μl 5% RBC for 1 h, or 30 min, respectively. Nanosponges were added at varying concentrations for dose-dependent experiments. At the end of infection, the plate was spun down at 3,000 × g for 5 min. The supernatant was collected and absorbance at 541 nm read.

### Mammalian Cell Cultures

A549 lung adenocarcinoma epithelial cells were cultured in RPMI with 10% heat-inactivated fetal bovine serum (HI-FBS) + 100 IU/mL penicillin/streptomycin (P/S). THP-1 human monocytes were cultured in RPMI with 10% HI-FBS + 100 IU/mL P/S. HEK-Blue-IL1β cells were cultured in Dulbecco's Modified Eagle Media (DMEM) with 100 IU/mL P/S, 100 μg/mL Zeocin, and 200 μg/mL Hygromycin B Gold. For colorimetric assays, media was free of phenol red. For infection assays, antibiotic-free media was used.

### Cell Viability Using ATP Based Luminescence Assay

Cell viability was measured using the CellTiter Glo ATP-based assay (Promega). A549 cells were seeded the day prior at 2 × 10^4^ cells/well in a 96-well plate, considering that the cell count roughly doubles with overnight incubation. THP-1 monocytes were differentiated using 25 μM PMA and seeded the day prior at 2 × 10^4^ cells/well in a 96-well plate. A549 and THP-1 were infected at MOI 20 and MOI 10, respectively. The plates were centrifuged at 500 × g for 5 min for bacteria contact with cells, then infected with live bacteria at 37°C for 2 h. When infecting with β-H/C+ extract, we used 5 μl and infect for 1 h. At the end of the experimental infection, the media was aspirated, and CellTiter Glo substrate added. The plate was shaken for 30 s to lyse cells and release ATP content, then allowed to incubate at room temperature for 10 min prior to reading luminescence.

### Cell Death Fluorescent Labeling With Propidium Iodide

A549 cells are seeded the day prior at 1 × 10^5^ cells in 500 μl media. THP-1 monocytes are differentiated with 25 μM PMA the day prior and seeded at 5 × 10^5^ cells in 500 μl media. Cell dishes used in these experiments are 35 mm glass-bottom, poly-L-lysine treated FluoroDish (World Precision Instruments). Cells were infected with GBS at MOI 10 for 2 h. At the end of infection, media was aspirated, and cells stained with 500 μl propidium iodide at a concentration of 0.5 μg/ml with 1% bovine serum albumin. Fluorescent microscopy was performed on the Zeiss Inverted Fluorescence microscope using the Red Alexa 594 protocol (618 nm).

### Human Neutrophil Isolation

Neutrophils are isolated using Polymorph Prep (Fresenius Kabi) as previously described (23). In summary, 30 ml human venous blood collected in heparinized tubes is carefully layered over 20 ml Polymorph Prep and centrifuged at 500 × g for 30 min at room temperature with brakes off. Two distinct white bands appear above the erythrocyte pellet, with the bottom white band containing neutrophils. The neutrophil layer was collected and pelleted at 630 × g for 8 min. Any remaining erythrocytes were lysed with hypotonic treatment using water with short, 30 s incubation periods. Neutrophil yield was counted using a hemocytometer.

### Neutrophil Killing Assay

GBS were grown overnight in liquid culture and subcultured to OD600 = 0.4. Neutrophils resuspended in RPMI (2 × 10^5^ cells) were added to a 96-well tissue culture plate and infected with GBS at MOI 1. The plates were centrifuged at 300 × g for 5 min for bacterial contact with cells and incubated at 37°C for 30 min. At the end of the infection, neutrophils were lysed by combining 20 μl of the sample to 180 μl water. Serial dilutions were plated on THB and incubated at 37°C overnight for CFU counting the next day.

### Inflammasome Production and Measurement

THP-1 cultured in RPMI + 10% FBS were differentiated with 25 nM PMA overnight at 2 × 10^5^ cells / well in a 12-well plate. HEK-Blue-IL1β reporter cells are seeded at 1 × 10^5^ cells / well in a 96-well plate the day prior. On the day of infection, PMA-containing media was aspirated and new media added to the THP-1 macrophages. The cells were then infected with live GBS at mid-log phase (OD600 = 0.4) at MOI 1 for 2 h at 37°C. After the infection, the THP-1 supernatant was transferred to the HEK-blue cells and incubated overnight at 37°C. On the following day, 50 μl HEK-blue supernatant was transferred to a new 96-well plate containing 150 μl/well SEAP detection reagent and allowed to develop at 37°C. Absorbance at 640 nm is read every 30 min.

### Human Protection

Healthy adult human blood donors were informed and consented under an approved UC San Diego Human Research Protections Program protocol (IRB #131002).

### Statistical Analysis

Data presented in this study are averaged values from three reads per sample, obtained from three replicates (each experiment done independently of each other) with standard deviation shown as error bars. Independent variables were analyzed using the Student's *t*-test. Experiments involving microscopic fluorescent images were done in triplicates, with a representative image shown in corresponding figures. Cell count involving microscopic images were completed using ImageJ (NIH and LOCI, University of Wisconsin). All experiment images were counted, values averaged, and results displayed in corresponding figures. All analyses were carried out using Prism (GraphPad, San Diego, CA). A *p*-value of ≤ 0.05 was deemed statistically significant.

## Results

### Human RBC-NS Do Not Directly Kill Nor Inhibit Growth of GBS

**Figure 2:**
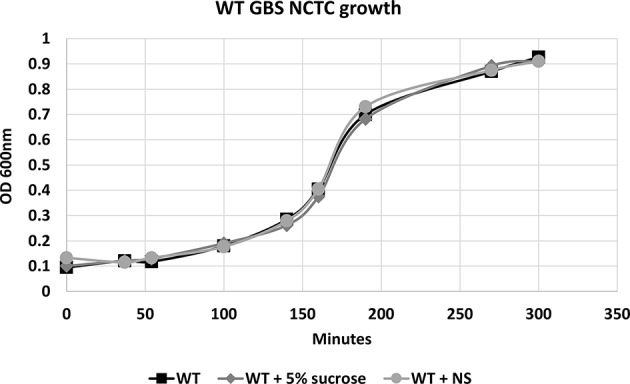
There is no difference in the growth pattern of WT NCTC GBS grown in THB, in THB + 5% sucrose, and in THB + hRBC-NS (in 5% sucrose). hRBC-NS does not have a direct inhibitory effect on the growth of GBS. Values averaged from three reads per sample, obtained from three independent replicates.

To confirm that hRBC-NS do not have direct effects on GBS growth or cell death, bacteria were grown in Todd Hewitt Broth (THB) with and without hRBC-NS. A five percent sucrose solution was tested in this experiment as a vehicle control as nanosponges are administered in a 5% sucrose solution. As demonstrated in Figure 2, no differences in growth patterns were observed with hRBC-NS exposure.

### Human RBC Nanosponges Reduce β-H/C Mediated Hemolysis

**Figure 3:**
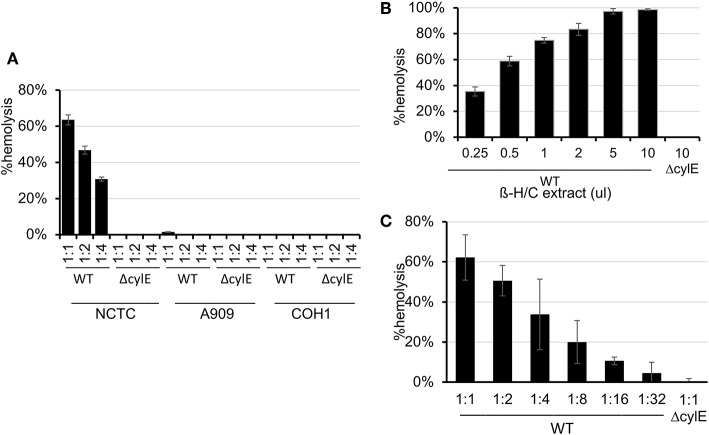
**(A)** The NCTC GBS strain demonstrated the greatest hemolytic activity compared to COH1 and A909. **(B)** Hemolytic activity was proportional to the concentration of bacterial supernatant containing stabilized β-H/C toxin in a dose-dependent manner, and **(C)** the concentration of bacteria. Values averaged from three reads per sample, obtained from three independent replicates. Values averaged from three reads per sample, obtained from three independent replicates.

**Figure 4:**
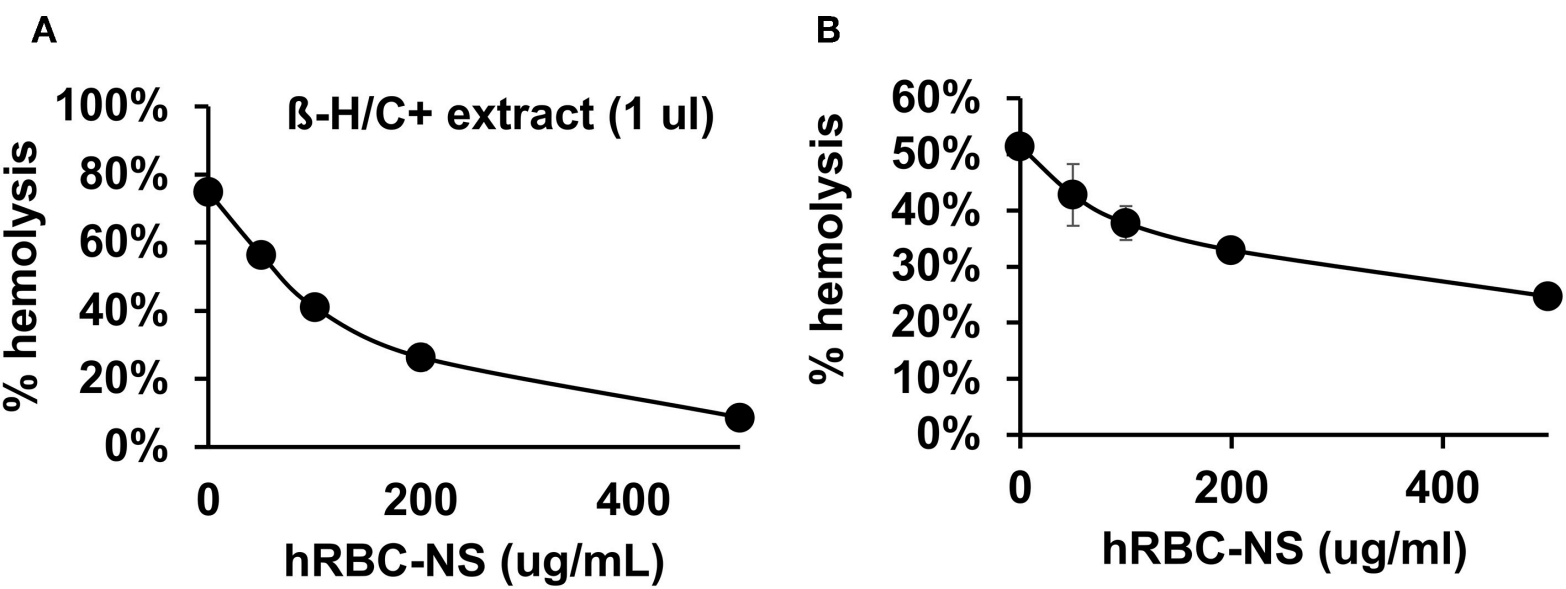
**(A)** hRBC-NS reduced hemolysis caused by β-H/C+ extract and **(B)** live NCTC GBS in a dose-dependent manner. Values averaged from three reads per sample, obtained from three independent replicates.

Because β-H/C is known for its signature hemolytic activity in the clinical laboratory, the ability of hRBC-NS to inhibit β-H/C-mediated hemolysis was evaluated as a first test of neutralization capacity. Well-characterized GBS clinical isolates including NCTC (serotype III), COH1 (serotype III), and A909 (serotype Ia) were used in human RBC lysis experiments. GBS strain NCTC demonstrated the greatest hemolytic activity compared to COH1 and A909, and was chosen as the primary GBS organism for subsequent experiments given it provided the strongest toxin challenge (Figure 3A). For all GBS strains, Δ*cylE* mutants lacking the encoding gene did not exhibit hemolytic activity. Hemolytic activity was proportional to the concentration of bacterial supernatant containing stabilized β-H/C toxin in a dose-dependent manner (Figure 3B), as well as concentration of bacteria (Figure 3C), with significant reductions in hemolysis achieved with concentration of hRBC-NS as low as 50 μg/ml. At a hRBC-NS concentration of 500 μg/ml, hemolytic activity resulting from 1 μl β-H/C extract and 4 × 10^6^ CFU live NCTC GBS declined from 75 ± 2% to 9 ± 3%, and from 51 ± 1% to 25 ± 1%, respectively (Figure 4).

### hRBC-NS Improve Lung Epithelial and Macrophage Cell Viability During GBS Exposure

**Figure 5:**
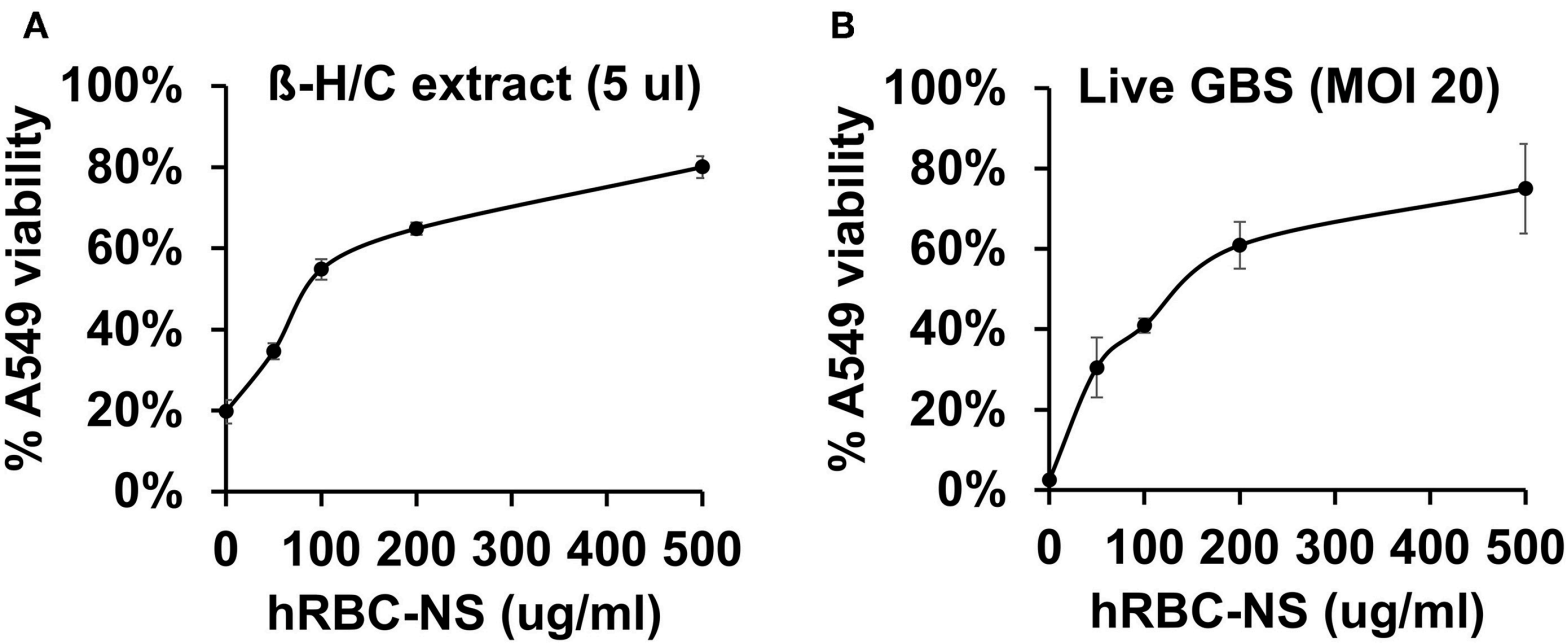
A549 cells infected by **(A)** β-H/C+ extract and **(B)** live NCTC GBS (MOI 20) have improved cell viability after treatment with hRBC-NS in a dose-dependent manner. Values averaged from three reads per sample, obtained from three independent replicates.

A549 lung adenocarcinoma cells closely resemble immature type II pulmonary epithelial cells and, therefore, provide an *in vitro* model of investigation to determine detrimental effects of β-H/C on preterm neonatal lung epithelium (24–26). Our results demonstrate reduced cytotoxicity of live NCTC GBS and β-H/C+ extract on A549 cells by hRBC-NS in a dose-dependent manner, and improved cell viability, as measured by ATP activity (Figure 5). After infection with β-H/C+ extract, A549 cell viability improved 4-fold following treatment with 500 μg/mL hRBC-NS (Figure 5A). Likewise, A549 viability increased 25-fold after infection with live GBS at MOI 20 (Figure 5B).

**Figure 6:**
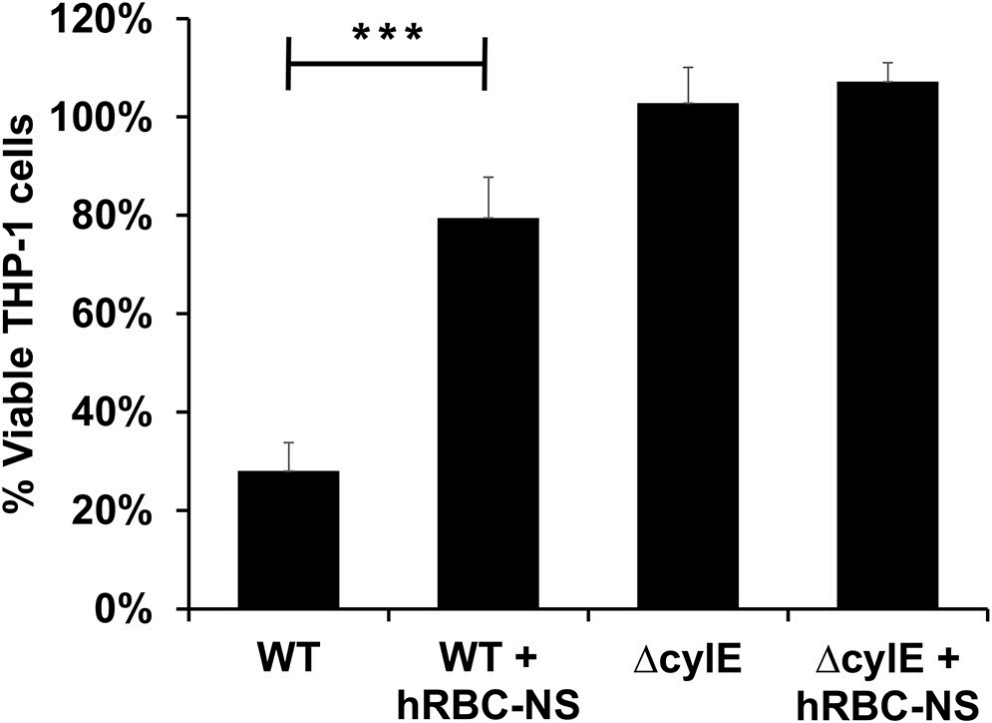
THP1 macrophages infected by live GBS at MOI 10 has improved cell viability after treating with hRBC-NS. ***p < 0.0001 compared to WT control. Values averaged from three reads per sample, obtained from three independent replicates.

GBS β-H/C induces macrophage apoptosis to enable host immune evasion (27–29). We, therefore, further examined the effects of β-H/C on THP-1 human-derived monocytes (30, 31). THP-1 cells were induced to differentiate into macrophages by exposing them to phorbol myristate acetate (PMA) (32), then employed as an *in vitro* model for GBS-induced macrophage cell death. GBS-infected THP-1 macrophages had markedly improved cell viability following hRBC-NS treatment, increasing roughly 3-fold over the WT control (Figure 6).

**Figure 7:**
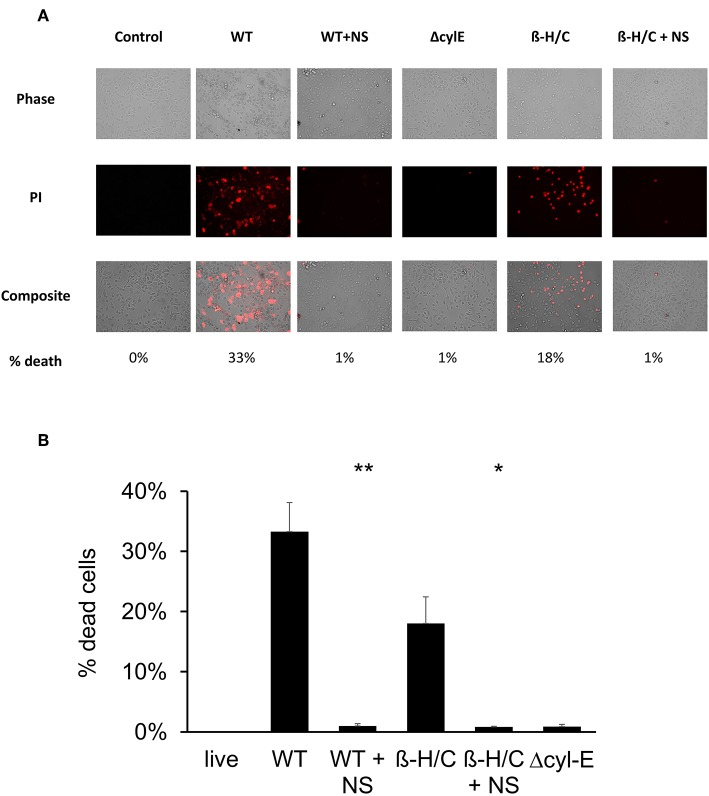
**(A)** Propidium iodide staining for dead cells. A549 cells were infected at 2 × 10^5^ cells/well with live GBS at MOI 10 for 2 h, or with β-H/C+ extract. Treatment with hRBC-NS at 500 μg/ml resulted in reduced A549 cell death compared to the control. Δcyl-E mutant GBS strain did not produce significant cell death. **(B)** Cell count of PI stained cells using FIJI/ImageJ demonstrates that hRBC-NS treatment reduces A549 cell death caused by infection with live WT GBS NCTC (**p = 0.0003) or with β-H/C+ extract (*p = 0.0026). Experiments performed in triplicate.

**Figure 8:**
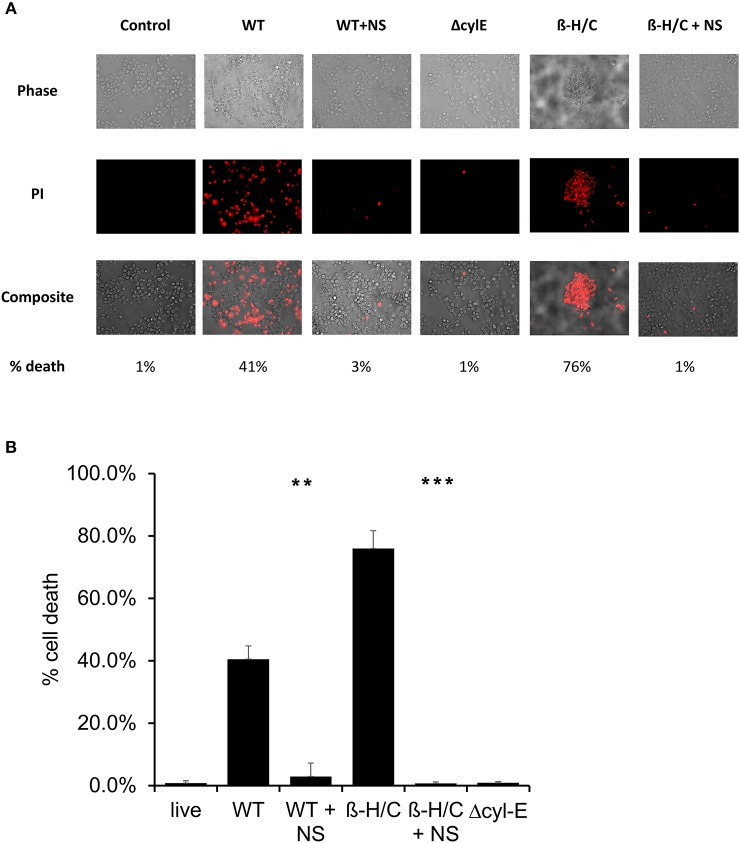
**(A)** Propidium iodide staining for dead cells. THP-1 cells were infected at 5 × 10^5^ cells/well with live GBS at MOI 10 for 2 h, or with β-H/C+ extract. Treatment with hRBC-NS at 500 μg/ml resulted in reduced THP-1 cell death compared to the control. Δcyl-E mutant GBS strain did not produce significant cell death. **(B)** Cell count of PI stained cells using FIJI/ImageJ demonstrates that hRBC-NS treatment reduces THP-1 cell death caused by infection with live WT GBS NCTC (**p = 0.0004) or with β-H/C+ extract (***p < 0.0001). Experiments performed in triplicate.

Cell death was also examined using fluorescent microscopy and propidium iodine, a DNA intercalating stain used as cell death marker. Both A549 lung epithelial cells (Figure 7) and THP-1 macrophages (Figure 8) demonstrated improved survival following NCTC GBS infection when treated with hRBC-NS. A549 cells were infected by live GBS at MOI 10 for 2 h, leading to death in one-third of the cells in the untreated group, but only a small fraction of cells (1 ± 0.4%) in the hRBC-NS treated group. A549 cells exposed to β-H/C+ extract had 18 ± 4% cell death, which declined to 1 ± 0.1% following hRBC-NS treatment. Similar results were observed with THP-1 macrophage cells, with infection with live GBS causing 41 ± 4% THP-1 cell death, but improved survival by 15-fold with hRBC-NS treatment. β-H/C+ extract-exposed THP-1 cells exhibited a high cell death rate of 76 ± 6%, which was greatly reduced following treatment with hRBC-NS (1 ± 0.4%).

### Neutrophil Killing Assay

**Figure 9:**
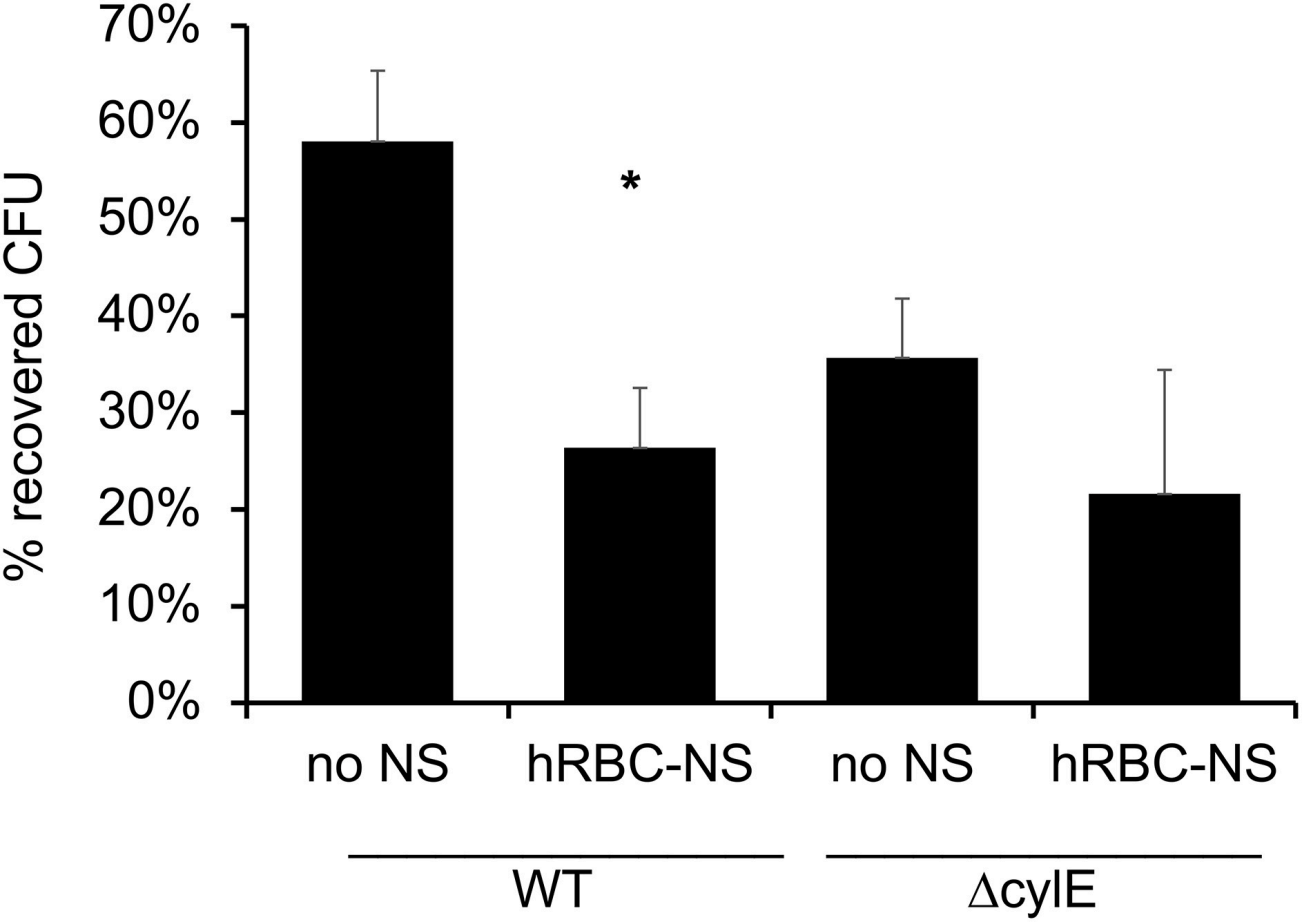
Neutrophils are exposed to live WT GBS and the surviving bacteria is plated for CFU quantification. The addition of hRBC-NS improves neutrophil killing of WT GBS (*p = 0.003). The absence of β-H/C does not lead to significant GBS killing, indicating that hRBC-NS does not have direct bactericidal effects. Values averaged from three reads per sample, obtained from three independent replicates.

It has been described that GBS β-H/C enables the bacteria to evade neutrophil extracellular traps and induce neutrophil cell death (33). In these experiments, we aimed to demonstrate that hRBC-NS can protect neutrophils from the cytotoxic effects of β-H/C and thus enhance the neutrophil-mediated killing of GBS. Human adult neutrophils, isolated on the day of the experiment, were stimulated with live NCTC GBS. hRBC-NS nearly doubled the neutrophils' ability to kill NCTC GBS bacteria (58 ± 7% vs. 26 ± 6 % CFU GBS, *p*-value of 0.0025; Figure 9). GBS Δ*cylE* mutants had low CFU recovery with and without hRBC-NS, and there was no statistically significant difference between the control and treatment groups. This data suggests the presence of β-H/C may suppress neutrophil killing of this GBS strain, but addition of hRBC-NS may enhance their bacterial clearance mechanisms.

### Macrophage Inflammasome Activation

**Figure 10:**
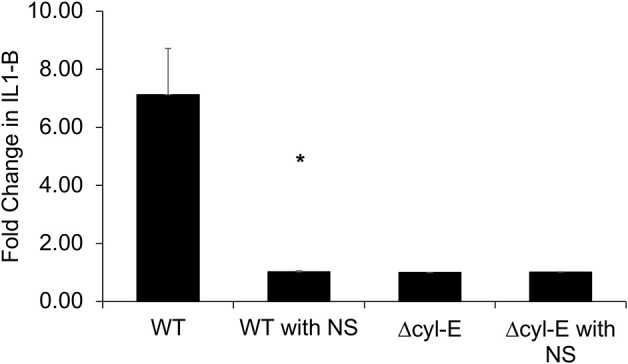
THP-1 cells were infected with GBS at MOI 1 for 2 h. The supernatant was collected and added to HEK-Blue-IL1β reporter cells to determine relative levels of IL-1β production. THP-1 macrophages infected by WT GBS exhibited increased production of IL-1β compared to uninfected controls (*p = 0.0027). Treatment with hRBC-NS reduced IL-1β production to baseline. Infection with GBS Δ*cylE* mutants did not lead to elevation in THP-1 macrophage IL-1β production. Values averaged from three reads per sample, obtained from three independent replicates.

NLRP3 inflammasome activation has been associated with β-H/C and identified as a crucial component of the human immune response to GBS (34, 35). In this experiment, THP-1 macrophages were infected with live GBS, and subsequent production of IL-1β is detected by HEK reporter cells that express IL-1 cell receptors. The HEK reporter cells, in turn, produce secreted embryonic alkaline phosphatase (SEAP), which is measured using a colorimetric assay. THP-1 macrophages infected by wild-type NCTC GBS exhibited a 7-fold increased production of IL-1β compared to controls. However, IL-1β production by GBS-infected THP-1 macrophages returned to baseline following treatment with hRBC-NS. Baseline IL-1β production was defined by SEAP production by HEK cells exposed to uninfected THP-1 cells. Infection with GBS Δ*cylE* mutants did not lead to elevation in THP-1 macrophage IL-1β production (Figure 10).

## Discussion

Pore-forming toxins, such as β-hemolysin/cytolysin of GBS, are the most abundant cytotoxic bacterial proteins and share a common function of perforating host cell membranes for bioactivity (22, 36). In general, disease burden and host responses are directly proportional to bacterial PFT secretion (8, 22). GBS-generated β-H/C is a critical virulence factor that induces apoptosis and necrosis of host epithelial and endothelial cells, thereby enabling microbial invasion, replication, and dissemination by way of immune defense subversion (8, 37). β-H/C also stimulates proinflammatory immune responses through NLRP3 inflammasome -dependent and -independent pathways (8), resulting in injury to professional phagocytes, neurons, and brain endothelial cells (37). Activation of p38 MAPK by β-H/C contributes to evasion of host defenses by GBS through induction of IL-10 expression and inhibition of macrophage activation (29). By breaching the blood brain barrier, β-H/C promotes the development of GBS meningitis, which is associated with long-term neurologic disabilities in almost half of affected neonates (8, 37) and non-pregnant adults (38). During pregnancy, β-H/C contributes to *in utero* infection, placental inflammation, preterm birth, fetal bacterial burden, and death (8, 37). β-H/C also mediates injury to lung epithelial and microvascular endothelial cells, the usual point of infection for neonatal EOS (8), with striking densities of GBS bacteria per gram lung tissue in primate pneumonia models (37).

**Figure 11:**
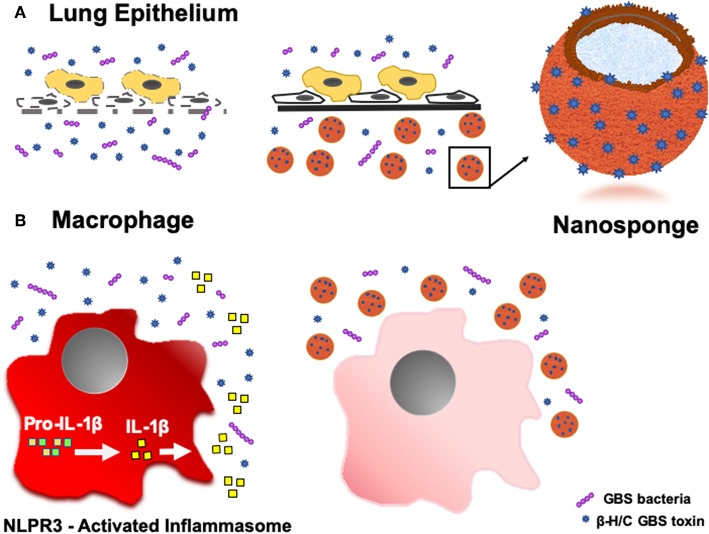
Schematic demonstrating the ability of hRBC-NS to sequester β-H/C to **(A)** reduce cytotoxic injury to lung epithelium and **(B)** attenuate macrophage inflammasome activation to inhibit IL-1β production.

Toxicity of PFTs has led to the development of various anti-virulence inhibitors, such as bacteriophages, immune modulating agents, prebiotics, monoclonal antibodies, small- molecular inhibitors, anti-sera, and exchange transfusions (39, 40), but these platforms typically require customized design for each different pathogenic toxin (22). Biomimetic nanosponges address these challenges by simply applying a hypotonic treatment to donor RBCs, then wrapping RBC membranes around polymeric cores via a nanoprecipitation method and sonication, thus converting the microscale RBC into a nanoscale “nanosponge,” measuring only 85 nm in diameter (18, 21, 41). This scale difference is advantageous for preferential toxin absorption, regardless of the PFT molecular structures and epitopic targets (22). This attraction may be due to: (a) drastic increases in the total number of circulating particles (i.e., one human RBC will provide enough membrane to prepare 40,000 hRBC-NS (22), (b) significant escalations in the frequency of collisions between the membrane substrate and the PFT, and/or (c) higher surface curvatures of the hRBC-NS compared to source RBC, providing increased surface tension and toxin-nanosponge affinity (22). By mimicking native RBCs, hRBC-NS bind PFTs, thereby diverting them away from their naturally intended cellular targets. Because β-H/C can cause host injury via diverse mechanisms, its noxious effects may be attenuated by non-specific nanosponges through a range of responses. That is, GBS β-H/C-induced epithelial cell injury and death involves nuclear chromatin clumping and programmed cell death, while β-H/C-mediated hemolysis results primarily from membrane cholesterol disruption (42). Once bound, nanosponges do not undergo hemolysis but rather lock in the toxins to keep them away host RBC membranes (18). The nanosponge construction also allows for long circulation half-life *in vivo* of ~40 h before clearance by hepatic macrophages, in addition to binding and retaining toxins more effectively than the host RBC membrane alone (41). For neonatal and adult GBS infection, hRBC-NS could potentially sequester β-H/C, reduce cytotoxic injury to lung epithelium, increase survival of macrophages and neutrophils, improve neutrophil killing of GBS organisms, and attenuate macrophage inflammasomes activation and production of IL-1β (Figure 11).

Nanoparticles are not only valuable as an adjuvant therapy to antimicrobials in the treatment of infection to prevent the onset of septic shock and multiple organ dysfunction syndrome (MODS), but they can also be engineered to facilitate the delivery of antimicrobials directly into cells and tissues used by organisms to evade host immune responses (43–45). In addition to hRBC-NS, which must be blood-typed to the recipient, endothelial, macrophage (46, 47), and platelet cell membrane engineered nanoparticles are also being developed, which may offer improved protection against Gram-negative bacteria and/or necrotizing enterocolitis. Macrophage-derived nanoparticles, for example, successfully attenuated proinflammatory responses and inhibit recruitment of excessive numbers of activated neutrophils to inflamed tissue sites, resulting in decreased tissue injury and reduced incidence of septic shock, MODS, and death in a murine model of *E. coli* sepsis (46). Nanoparticles are biocompatible and biodegradable in the liver, with *in vivo* animal studies demonstrating absence of liver tissue or Kuffer cell injury (18). Moreover, hRBC-NS do not directly engage in bacterial cycle disruption or activities that may elicit resistance when compared with traditional antibiotics. This is an important nanoparticle characteristic, since the proportion of GBS isolates with *in vitro* resistance to clindamycin or erythromycin have steadily increased (1, 5).

Red blood cell-membrane coated PLGA nanoparticles have a promising role in broadly treating a number of ailments, ranging from infections to autoimmune diseases (48) and hemolytic diseases (49). Determining *in vivo* efficacy of nanosponges as adjuvant therapies in murine GBS models of neonatal pneumonia and adult sepsis are important next steps toward clinical implementation of this innovative therapeutic. Multiple studies are currently ongoing to evaluate their role in infection, necrotizing enterocolitis, cancer, autoimmune disease, and hemolytic conditions with cautious optimism.

## Conclusion

We demonstrated that hRBC-NS attenuates β-H/C-mediated hemolysis, lung epithelial cell death, macrophage apoptosis, suppression of neutrophil bactericidal properties, and inflammasome activity. This proof-of-principle study demonstrates that toxin hRBC-NS neutralization may provide a new avenue for adjunctive treatment in neonatal GBS sepsis by sequestering and inhibiting β-H/C activity.

## Data Availability Statement

The datasets generated for this study are available on request to the corresponding author.

## Ethics Statement

The studies involving human participants were reviewed and approved by UC San Diego Human Research Protections Program protocol (IRB #131002). The patients/participants provided their written informed consent to participate in this study.

## Author Contributions

SL and VN conceived of the study. JK and TE performed experiments. LZ engineered the nanosponges. JK, SL, and VN drafted manuscript, which all authors critically reviewed. All authors designed experiments and analyzed data.

### Conflict of Interest

The authors declare that the research was conducted in the absence of any commercial or financial relationships that could be construed as a potential conflict of interest.
